# Silver hair in a neonate: a tale of 2 fatal cases

**DOI:** 10.1093/omcr/omae106

**Published:** 2024-09-12

**Authors:** Lakshmi Satish Kumar, Prashanth Ranya Raghavendra, Sruthi Nair, Muthu Vijaya Nathan D, Umair Ahmed Bargir, Anitha Haribalakrishna, Sunanda Arun Mahajan

**Affiliations:** Department of Neonatology, Seth G.S. Medical College and King Edward Memorial Hospital, Acharya Donde Marg, Parel, Mumbai 400012, India; Department of Neonatology, Seth G.S. Medical College and King Edward Memorial Hospital, Acharya Donde Marg, Parel, Mumbai 400012, India; Department of Neonatology, Seth G.S. Medical College and King Edward Memorial Hospital, Acharya Donde Marg, Parel, Mumbai 400012, India; Department of Neonatology, Seth G.S. Medical College and King Edward Memorial Hospital, Acharya Donde Marg, Parel, Mumbai 400012, India; Department of Pediatric Immunology and Leukocyte Biology, ICMR-National Institute of Immunohaematology, Seth G.S. Medical College and King Edward Memorial Hospital, Acharya Donde Marg, Mumbai, Maharashtra 400012, India; Department of Neonatology, Seth G.S. Medical College and King Edward Memorial Hospital, Acharya Donde Marg, Parel, Mumbai 400012, India; Department of Dermatology, Seth G.S. Medical College and King Edward Memorial Hospital, Acharya Donde Marg, Parel, Mumbai 400012, India

**Keywords:** Griscelli syndrome, silver hair, immunodeficiency disorders

## Abstract

Silver hair in a neonate is an uncommon occurrence. The aetiology of this condition is varied and is associated with immunodeficiency disorders such as Griscelli syndrome and Chédiak-Higashi syndrome. A preterm neonate with Griscelli syndrome type 2 might present with just silver colour staining of hair including the lanugo hair with no other complications. In those with associated systemic abnormalities such as congenital pulmonary airway malformation, further evaluation for conditions such as Menke-Kinke hair syndrome is required. In this case series, we describe two unique cases of silver hair syndrome in preterm neonates with their clinical description, course in the hospital, role of hair mount and genetic testing for further identification and diagnosis of this disorder.

## Introduction

Silver hair syndrome in a neonate might be a unique presentation of an underlying serious systemic illness and the exact presentation of these syndromes in the neonatal period remains less known. Those presenting in the neonatal period are often diagnosed and worked up for aetiology once upon the development of extra dermatological complications. Griscelli syndrome (GS) is one such rare inherited disease characterized by neonatal silver hair and immunodeficiency. A total of 769 cases of silver hair have been reported till date [1].

## Case report 1

A 1310 g female neonate born at 29 weeks 5 days of gestation as a dichorionic diamniotic twin to a second-degree consanguineous married couple. She was born with abnormally light and silvery hair over the head and body. On further examination, the hair was silver-coloured both at the root and the tip, distributed uniformly across the head and body, with the rest of the examination being normal including a normally pigmented iris and neurological exam.

There was a family history of an elder sibling currently 11 years old and attending special school, also being born with silver hair, and had neuro-regression from two years of age. She was diagnosed with Griscelli Syndrome with a pathogenic homozygous nonsense variant in exon 7 of the *RAB27A gene* [c.550C > T], p.R184X. She underwent hematopoietic stem cell transplantation at the age of 5 years. The parents did not undergo any genetic testing, or any additional prenatal tests offered in this pregnancy. The other twin had a normal physical examination at birth. At admission to the neonatal intensive care unit (NICU), she had respiratory distress syndrome requiring respiratory support till day 7 of life and was on breastmilk feeds throughout. The complete blood count, peripheral blood smear, C-reactive protein, blood culture, liver and renal function tests were normal.

The hair mount and light microscopy of silver hair revealed scattered large irregular melanin pigments distributed in a non-uniform manner with no clumping (Image 4A). Sanger sequencing identified the homozygous nonsense variant in exon 7 of the *RAB27A gene.* She was diagnosed with Griscelli’s syndrome upon clinical and genetic confirmation and was discharged from the hospital on day 33 of life.

She developed an acute onset of respiratory distress secondary to pneumonia on day 40 of life and was readmitted with respiratory failure. Blood tests done during the same period revealed late-onset sepsis and the infant succumbed within 24 h of readmission.

## Case report 2

A 1036 g female neonate was born at 28 weeks to a non-consanguineously married couple. The mother was referred to us in preterm labour. A scan done at 24 weeks of gestation showed Congenital Pulmonary Airway Malformation (CPAM) of the left lung with associated polyhydramnios. No intervention or genetic testing was done during this pregnancy.

At birth, she required resuscitation and was started on mechanical ventilation for left-sided CPAM. She was born with silver-coloured lanugo hair showing depigmentation from root to tip of the hair distributed uniformly across the entire torso and back. They were not brittle, with no wiring or kinky appearance. The rest of the general and systemic examination was normal with no dysmorphism, normally pigmented iris and neurological exam (Image 2A and B).

**Image 1:**
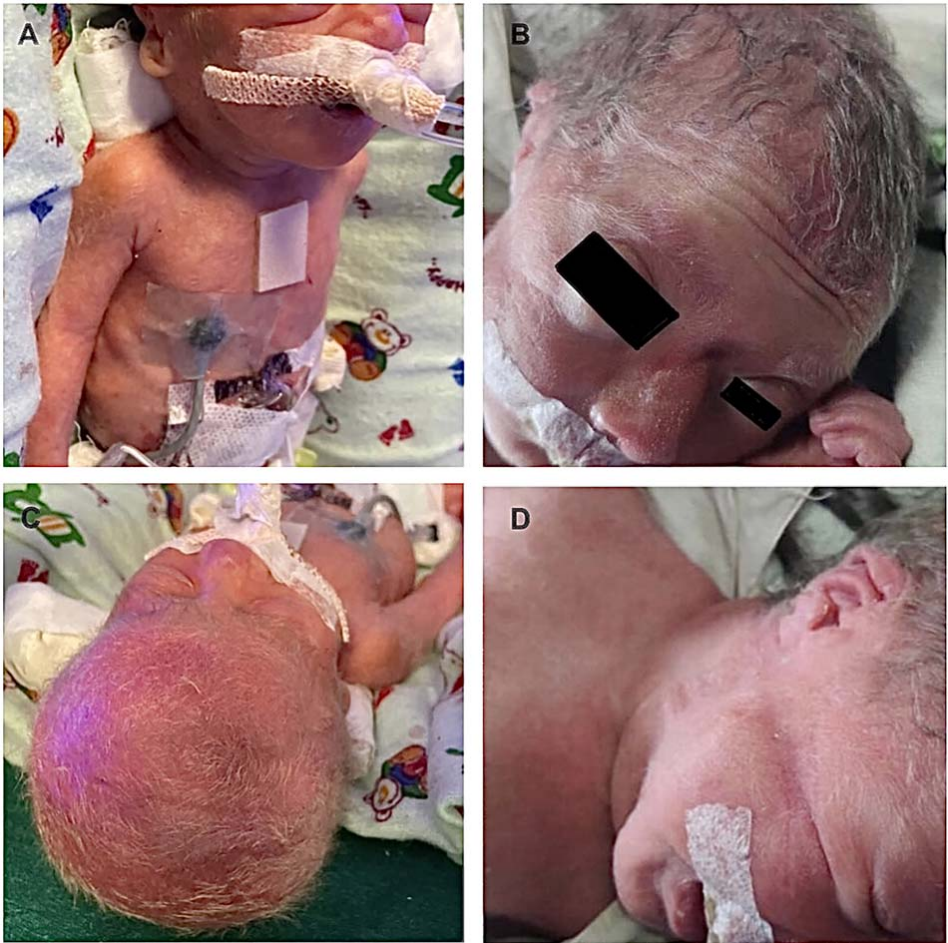
(**A–D**) of case report 1. All show silvery white hair on the scalp and the rest of the body from different views present.

**Image 2:**
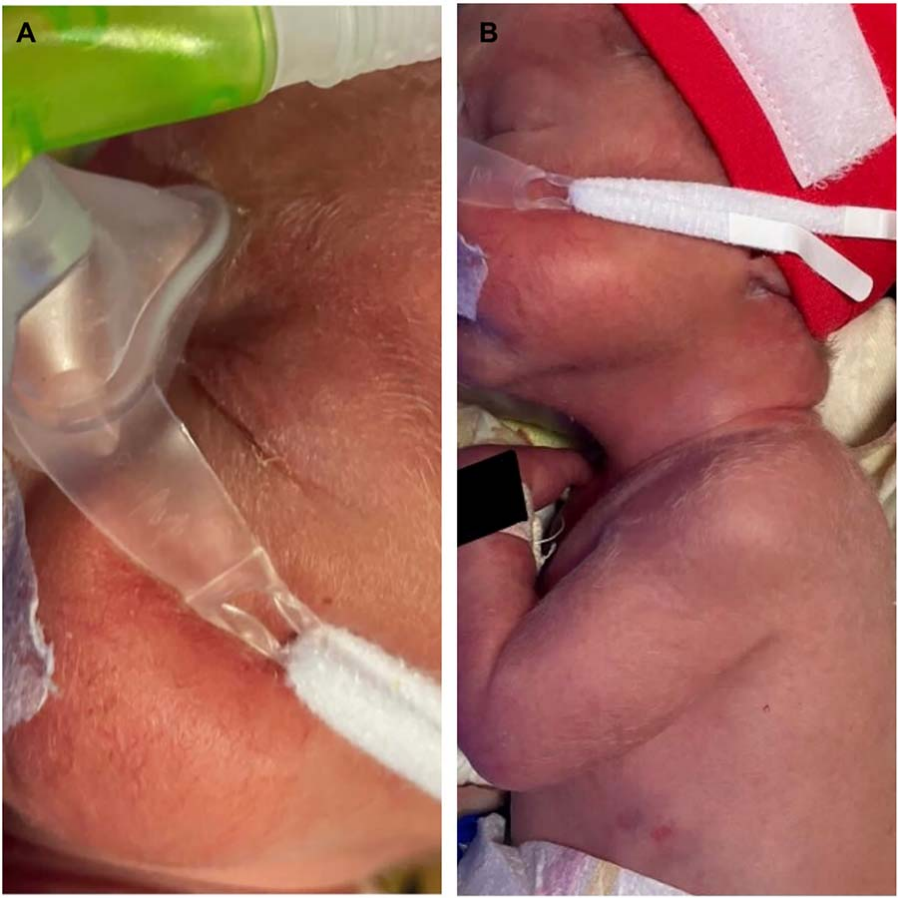
(**A** and **B**) of case report 2. (**A**) Silver-white staining of lanugo hair noted over the face. (**B**)Silver-white hair noted over upper back and arm.

**Image 3:**
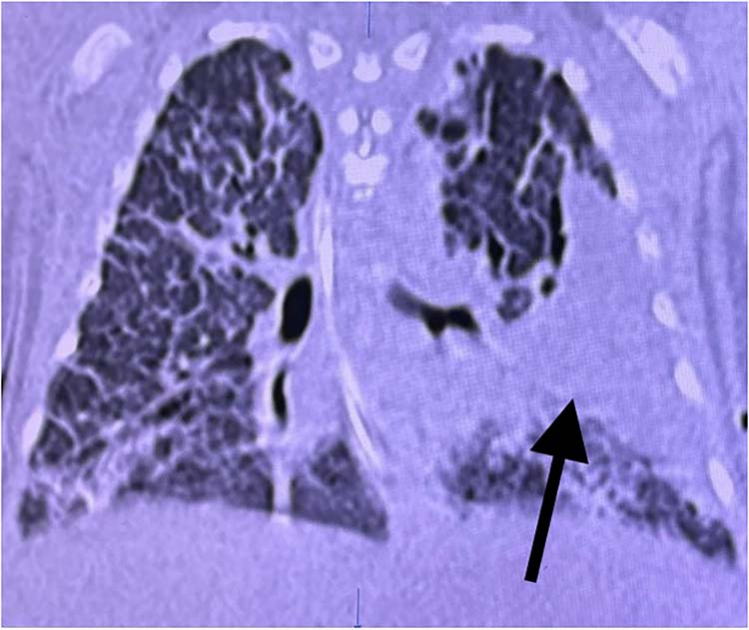
A chest CT image (of case report 2) ill-defined isolated cystic fluid-filled area in the left upper and lower lobe suggestive of CPAM (black arrow).

Initial blood investigations for neonatal sepsis were negative and peripheral blood smear, coagulopathy screen, liver and renal function tests were normal. X-ray and high-resolution computed tomography confirmed left-sided CPAM (Image 3), which in the presence of silver hair led to the probable diagnosis of Menke-Kinky syndrome or Griscelli syndrome.

**Image 4:**
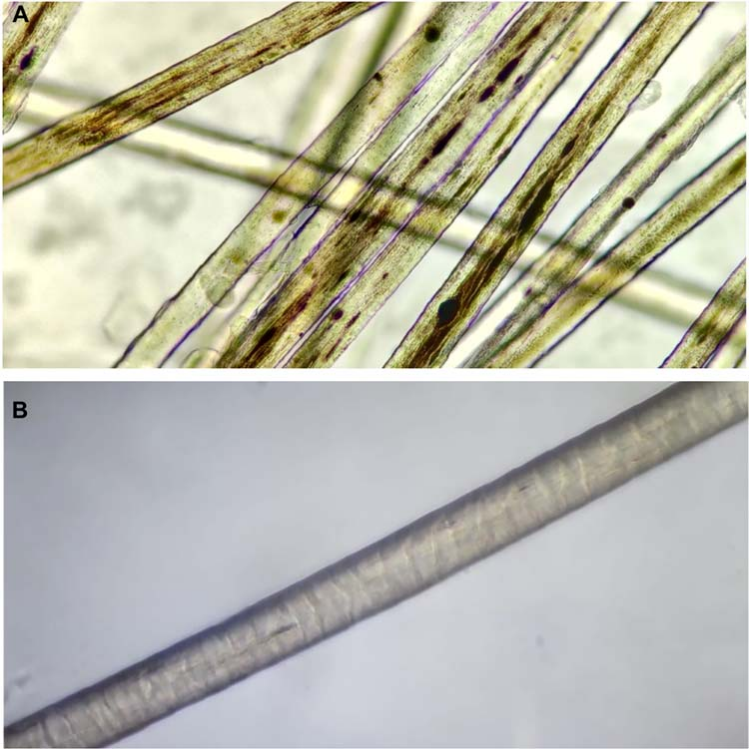
Hair mount images (**A**): Scattered large irregular melanin pigments distributed in a non-uniform manner-from case report 1. (**B**) Hair mount and light microscopy showing scanty melanin pigments with no clumping-from case report 2.

Hair mount and light microscopy revealed scanty melanin pigments distributed in a non-uniform manner with no clumping (Image 4B). There was no evidence of pili torti, trichorrhexis nodosa or fragmentation of hair. Genetic analysis could not be performed due to financial constraints. The exact cause of silver hair syndrome in this neonate could not be confirmed and was attributed to probable Griscelli syndrome after ruling out other probable differentials and reassessment of the hair mount. She succumbed to a worsening respiratory condition secondary to CPAM and ventilator-associated pneumonia.

## Discussion

Griscelli syndrome (GS) is a rare inherited disease which presents with very subtle albinism at earlier stages of life [2]. This syndrome is an autosomal recessive disorder with homozygous or compound heterozygous mutations in the *RAB27A* gene [3]. A silvery-white colour of hair, alternating pattern of clumps of melanocytes with periods of depigmentation, along with other signs like pancytopenia, decreased proteins, triglycerides, fibrinogen, and gamma globulins are seen consistently [4].

**Table 1:** Showing age, ethnicity, clinical features and outcomes of the reported cases of neonatal presentation of Griscelli syndrome [7–10]

Case No	Age at presentation	Ethnicity	Type of Griscelli syndrome	Clinical features at presentation	Outcome
1.	13 days old male baby (presentation since birth)	Indian	Griscelli syndrome type II(GS II)	Extremely fair skin, silvery gray scalp hair,white eyelashes, eyebrows,body hair.	Child is currently aged one year and on regular follow up. Normal motor and mental development for his age. Bone marrow transplantation being considered [7].
2.	5 year old girl (presentation since birth)	Danish	GS II	Unusual silver blond hair.	Successful stem cell transplantation at 6 months of age [8].
3.	6 yr old boy (presentation from infancy)	Afghan	GS II	Slightly grey hair from infancy. Stroke like attack, squinting of eyes, speech difficulty and hemiplegia since 3 yrs of age, progressive fever episodes and hepatosplenomegaly since 6 yrs of age.	Received Bone marrow transplantation, child has delayed development and hemiplegia [8].
4.	Twin A-3 yrs of age Twin B-3 yrs and 9 months of age	Not known	GS II GS II	Encephalitis like presentation Fever associated with axial and appendicular ataxia, light silvery grey colored scalp hair, late episodes of thrombocytopenia, hepatosplenomegaly and coma.	Death [9]. Death [9].
5.	2.5 yr old male	Indian	GS II	Progressive abdominal distinction, pallor and recurrent fever episodes since 1 year of age, on and off ear discharge, history of 4 blood transfusions, grossly malnourished, silvery grey scalp hair, white eyelashes, sparse eyebrows, pedal edema, firm hepatomegaly.	Not known [10].

**Table 2:** Showing the various clinical conditions presenting with silver hair along with respective hair manifestations, skin presentations and hair mount examination findings through light microscopy method [11–15]

Clinical Condition	Hair manifestation	Skin Manifestation	Hair Mount examination
1. Griscelli’s syndrome type—I	Silvery grey hair	Fair skin	Large and irregular medullary clumps of pigments, road dividing line- like appearance [11].
2. Griscelli’s syndrome type—II	Silver grey scalp and body hair, white to grey eyelashes.	Extremely fair skin	Irregularly placed clumps of pigments, similar to type I: road dividing line-like appearance [11].
3. Griscelli’s syndrome type—III	Hypopigmentation of hair	Hypopigmentation of skin (skin findings more prominent than that of hair in this type).	Irregularities in pigment distribution (not as prominent as Griscelli’s syndrome type I and II) [12].
4. Hermansky Pudlak disease	Hair colour varies from white to brown.	Skin colour ranges from white to olive, or at least a shade lighter than that of family members.	Pigmentary dilution, giant melanosomes with clumping, very scanty and scattered pigments [13].
5. Chediak Higashi’s syndrome	Partial. albinism, slivery grey hair.	Hyperpigmentation in sun—exposed areas.	Giant melanosomes, regularly placed pigmentation (homogenous distribution) with clumping, irregular hair shaft thickness, hypo-pigmentation with areas of normal pigmentation [14].
6. Menke Kinky Hair	Normal to hypopigmentation of scalp hair including eyebrow hair.	Doughy skin, lighter in colour.	Clumping of melanin along hair shafts, pili torti, monilethrix, fragmentation, trichorrhexis nodosa, trichoclasis [15].

Three distinct forms of Griscelli syndrome exist. Type I results from mutations in the *MYO5A* gene and is characterized by primary neurological impairment including seizures and hypotonia, while immune-related features like susceptibility to infections and hemophagocytic syndrome (HLH) are not typically observed. Type II (GS II), arises from mutations in the *RAB27A* gene and lacks primary neurological manifestations. It is associated with uncontrolled activation of T lymphocytes and macrophages, often leading to HLH [5]. Type 3 tends to have a good prognosis and does not present with immuno-neurological features [6]. Table 1 shows similar cases of Griscelli syndrome with neonatal onset [7–10].

GS II is most commonly diagnosed between 4 months and 7 years with type 3 usually being diagnosed by 10 years [9]. Clinical presentation in these 2 preterm neonates has shown even the lanugo hair can be involved. There was no evidence of neurological involvement in them and the cause of death in these cases was due to underlying prematurity and associated congenital lung malformation. There were no features suggestive of HLH and immunodeficiency reemphasising that these complications occur in later infancy.

Neonatal presentation of silver hair syndrome is indeed atypical with predominantly abnormal hair and other complications developing over time. Clinical examination by a trained clinician and confirmation of the same with a pediatric dermatologist is of utmost significance in diagnosing these conditions in which genetic diagnosis is not readily available or the neonate succumbs to illness before the genetic testing. The key clinical and skin biopsy features of each of these silver hair syndromes are summarized in table 2.

Findings in the index neonate reported as case 2 included silvery coloured lanugo hair, scalp and eyebrow hair showing depigmentation and scanty melanin pigments, however with the absence of clumping on light microscopy.

Microscopic examination of the hair shaft in GS reveals the presence of scanty but larger melanin granules in the hair shafts compared to normal hair. The difference between type 1 & GS II is in the clumping of melanin granules which is absent in GS II. Irregularly clustered abnormal discrete conglomerations of melanin pigment throughout the interior portion of the shaft are seen in type 3 [7]. Desirable treatment for Griscelli syndrome type 2 has by far been a Hematopoietic Stem Cell Transplantation (HSTC) for those cases with an associated genetic linkage [7, 8].

The merits of this case series are the diagnosis based on clinical examination which correlates with the hair mount findings and early recognition in the neonatal period enables better prognostication and parenteral preparedness. The limitation of the second case is the probable diagnosis of GS without genetic confirmation. The authors would like to highlight here that a careful interpretation of clinical signs and hair mount by a trained clinician helps to narrow down the differentials of this rare condition and needs confirmation by genetic diagnosis as in the first case, for better diagnostic accuracy.
